# Supplementary material for: Multiplex Analysis of CircRNAs from Plasma Extracellular Vesicle-Enriched Samples for the Detection of Early-Stage Non-Small Cell Lung Cancer
Source: Pharmaceutics. 2022 Sep 24;14(10):2034. doi: 10.3390/pharmaceutics14102034 (PMC9610636; doi:10.3390/pharmaceutics14102034)
Supplement: Supplementary file 1 [file pharmaceutics-14-02034-s001.zip › pharmaceutics-1881461-supplementary.pdf]

Supplementary data for

Study of circRNAs from plasma extracellular vesicle-enriched samples for early-stage non-small cell lung cancer detection using a multiplex hybridization-based method

Carlos Pedraz-Valdunciel, Stavros Giannoukakos, Ana Gimenez-Capitán, Diogo Fortunato, Martyna Filipska, Jordi Bertrán-Alamillo, Jillian W.P. Bracht, Joselyn Valarezo, Natasa Zarovni, Alberto Fernández-Hilario, Michael Hackenberg, Andrés Aguilar-Hernández, Miguel Angel Molina-Vila, Rafael Rosell

Correspondence to: cpedraz@panoncology.com / carlospedraz@icloud.com / rrosell@iconcologia.net

Supplementary Tables

Table S1. CircRNAs detected in the different plasma volumes of the same patient with 14 and 20 cycles of pre-amplification.

		500 µL				1000 µL				1500 µL			
		14 cycles		20 cycles		14 cycles		20 cycles		14 cycles		20 cycles	
		R1	R2	R1	R2	R1	R2	R1	R2	R1	R2	R1	R2
Detected circRNA (counts > 0)		31	24	38	28	29	25	15	16	8	15	16	25
Detected circRNA (counts > 10)		20	18	33	24	9	5	4	0	0	1	1	5

R= replica

Table S2. CircRNAs detected in the plasma of the same individual subjected to 10, 12 and 14 pre-amplification cycles.

	500 µL					
	10 cycles		12 cycles		14 cycles	
	R1	R2	R1	R2	R1	R2
Detected circRNA (counts > 0)	58	45	58	47	71	47
Detected circRNA (counts > 10)	38	32	39	41	48	40

R= replica

Table S3. circRNAs identified in early-state NSCLC and non-cancer control cohorts.

circRNAs expressed only in the NSCLC cohort	circRNAs expressed in NSCLC and control cohorts	circRNAs expressed only in the control cohort
circTMEM39B, circZFR, circRHD, circPIK3C2B	*circHOMER1, circFARSA, circHIPK3, circRUSC2, circC1orf116, circAHNAK, circBACH2, circEPB41L2, circSEMA5A, circCHD1L, circPIK3R1, circADAM22, circDENND1B, circFUT8, circDUS2L, circRANGAP1, circPDE5A, circSNX25, circNUPL2, circCSPP1, circCOL11A1, circNEDD4L, circNEDD4L-2, circUHRF1, circTASP1, circAASDH, circMYBL1, circB4GALT2, circCHST15, circCORO1C, circVRK1, circMGA, circGAS8, circSMAD2, circSLC8A1, circPMS1, circCCDC134, circFOXP1, circUBXN7, circSMARCA5, circCCNB1, circFAM13B, circC1GALT1, circRUNX1, circLIN54, circSND1, circCLK1, circHIBADH, circCHN1, circLYPLAL1, circPSD3, circSOX13, circRDH11, circYWHAZ, circDNA2, circANXA7, circZCCHC6, circTXNDC11, circDHCR24, circACP6, circUSP3, circCHD2, circITGAX, circBANP, circACACA, circBNC2*	*circNOL6, circNUP98*

Table S4. Normalized counts of differentially expressed circRNAs found in the early-stage NSCLC cohort.

circRNA	Gene	Mean non-cancer cohort	Mean early-stage NSCLC cohort (controls)	t-test	Fold change
circRNA_HIPK3	HIPK3	161	361	0.042	2.24
hsa_circRNA_001640	EPB41L2	109	230	0.002	2.11
hsa_circRNA_100421	DENN1B	27	62	0.049	2.31
hsa_circRNA_103809	ZFR	0.41	4	0.014	10.98
hsa_circ_0001495	CCNB1	7	21	0.040	3.10
hsa_circ_0001675	C1GALT1	1	7	0.009	10.58
hsa_circ_0007037	ZCCHC6	54	138	0.039	2.57
hsa_circ_0035654	USP3	3	22	0.016	7.13

NSCLC= non-small cell lung cancer

<u>**Supplementary Figures**</u>

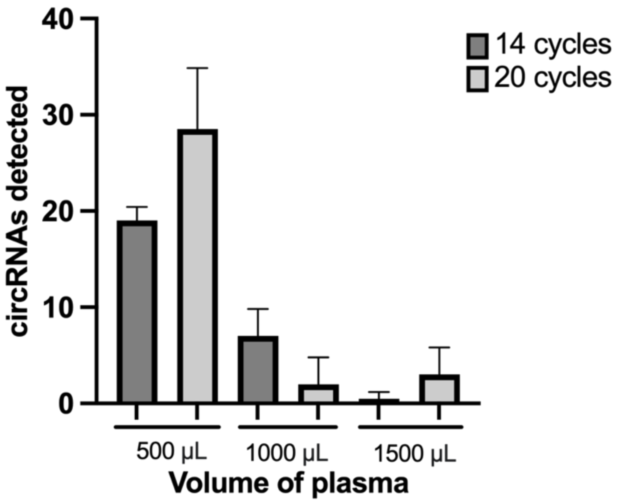

Figure S1. Plasma input testing. Number of circRNAs with a score > 10 counts after background removal for each of the volumes tested. Error bars indicate standard deviation.

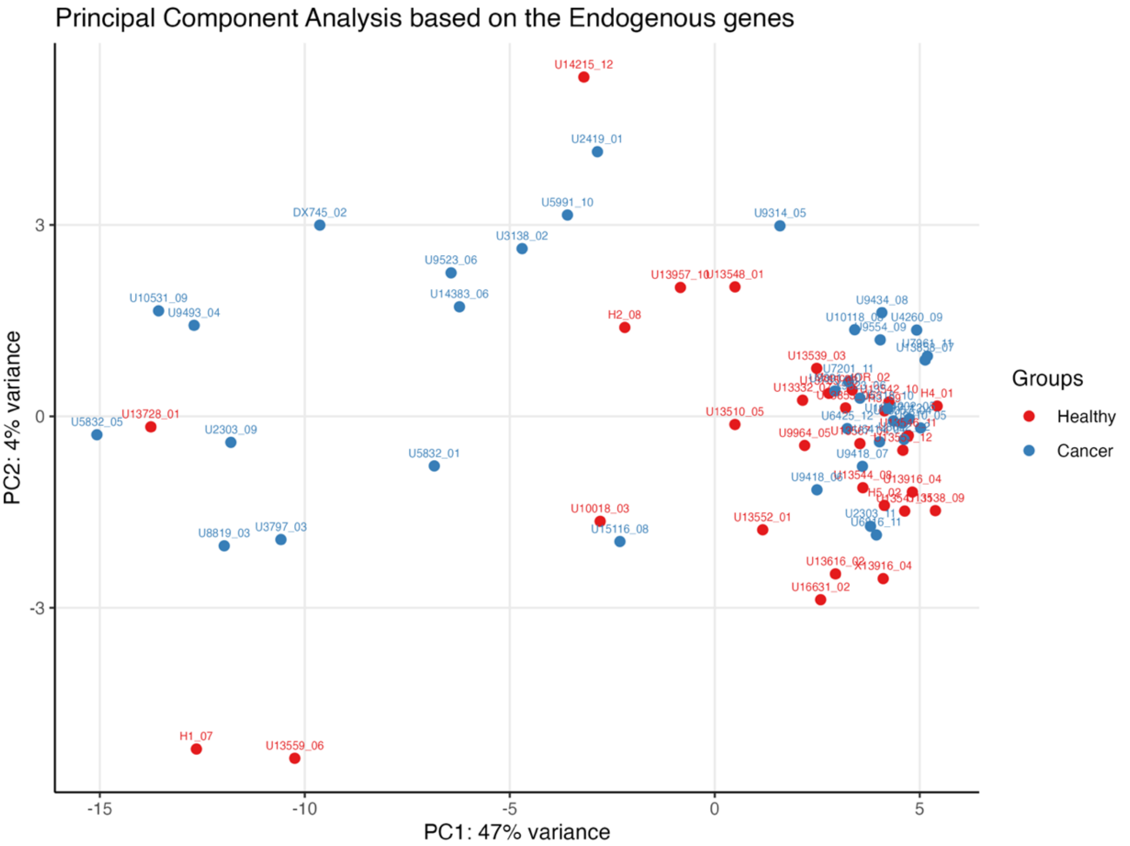

Figure S2. Principal Component Analysis of the transformed raw data. Outliers highlighted by interquartile range plot analysis were also found on the far-left side of the plot.

A

B

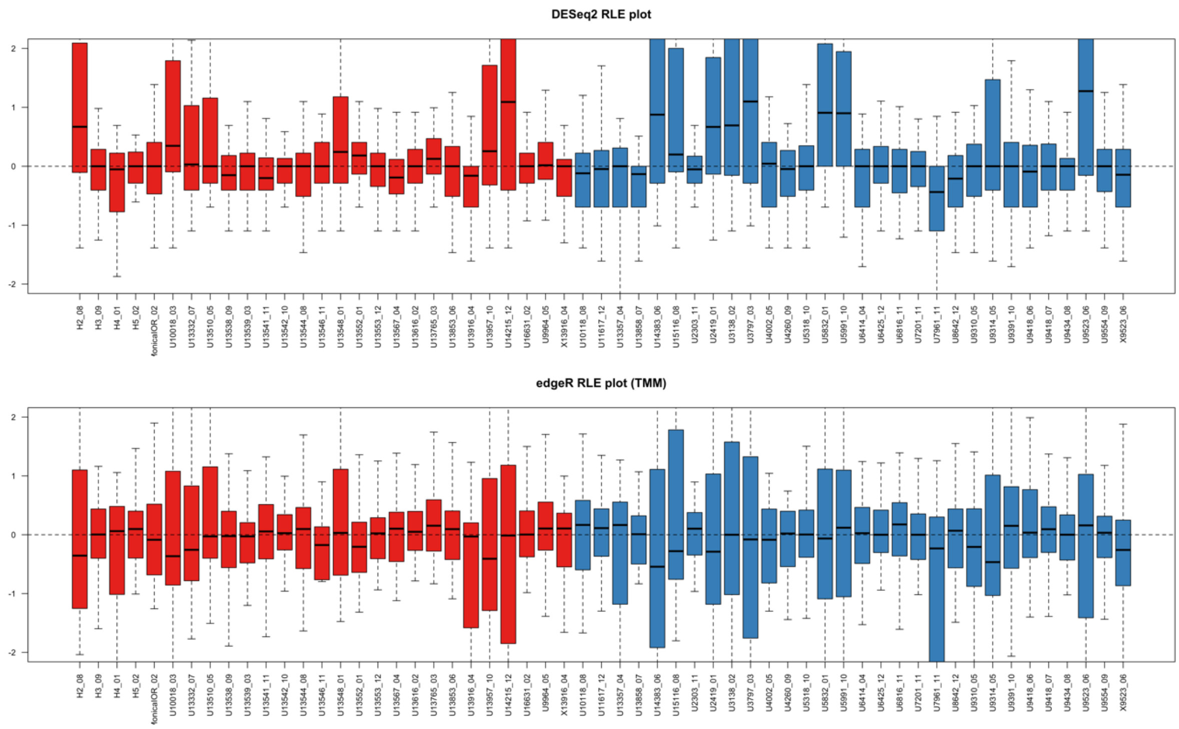

C

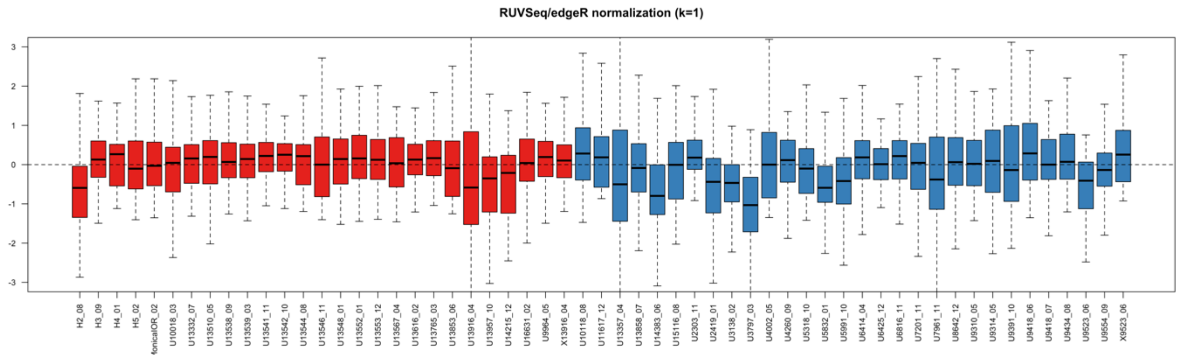

Figure S3. Assessment of the different normalization processes by RLE plot analysis. **(A)** RLE plot of normalized counts by DESeq2. **(B)** RLE plot of normalized counts by edgeR. **(C)** RLE plot of normalized counts using a combination of the edgeR-RUVg methods (k = 1).

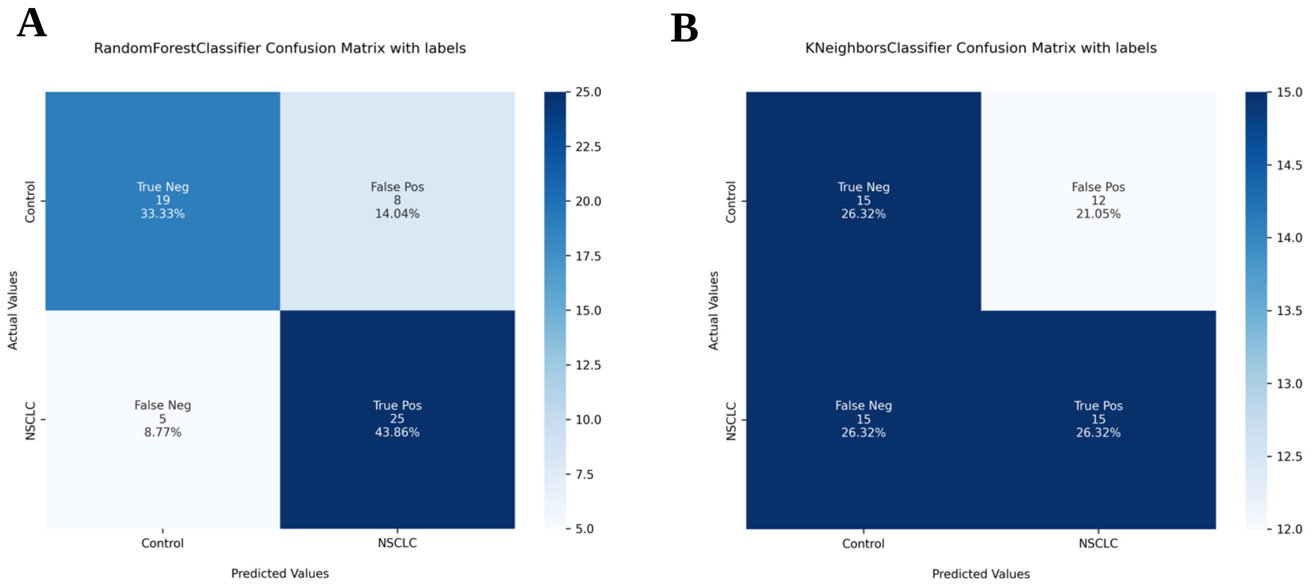

Figure S4. Confusion matrices summarizing the performance of the different classification algorithms. (**A**) Confusion matrix of the of the RFF classifier. (**B**) Confusion matrix of the KNN classifier. In both cases, 5-Fold CV was used.

RF = Random Forest, KNN = K-Nearest Neighbor, 5-CV = 5-Cross Validation, RFE = Recursive Feature Elimination.
